# FtsZ Placement in Nucleoid-Free Bacteria

**DOI:** 10.1371/journal.pone.0091984

**Published:** 2014-03-17

**Authors:** Manuel Pazos, Mercedes Casanova, Pilar Palacios, William Margolin, Paolo Natale, Miguel Vicente

**Affiliations:** 1 Centro Nacional de Biotecnología - Consejo Superior de Investigaciones Científicas (CNB-CSIC), Madrid, Spain; 2 Department of Microbiology and Molecular Genetics, University of Texas Medical School at Houston, Houston, Texas, United States of America; University of Massachusetts Medical School, United States of America

## Abstract

We describe the placement of the cytoplasmic FtsZ protein, an essential component of the division septum, in nucleoid-free *Escherichia coli* maxicells. The absence of the nucleoid is accompanied in maxicells by degradation of the SlmA protein. This protein, together with the nucleoid, prevents the placement of the septum in the regions occupied by the chromosome by a mechanism called nucleoid occlusion (NO). A second septum placement mechanism, the MinCDE system (Min) involving a pole-to-pole oscillation of three proteins, nonetheless remains active in maxicells. Both Min and NO act on the polymerization of FtsZ, preventing its assembly into an FtsZ-ring except at midcell. Our results show that even in the total absence of NO, Min oscillations can direct placement of FtsZ in maxicells. Deletion of the FtsZ carboxyl terminal domain (FtsZ*), a central hub that receives signals from a variety of proteins including MinC, FtsA and ZipA, produces a Min-insensitive form of FtsZ unable to interact with the membrane-anchoring FtsA and ZipA proteins. This protein produces a totally disorganized pattern of FtsZ localization inside the maxicell cytoplasm. In contrast, FtsZ*-VM, an artificially cytoplasmic membrane-anchored variant of FtsZ*, forms helical or repetitive ring structures distributed along the entire length of maxicells even in the absence of NO. These results show that membrane anchoring is needed to organize FtsZ into rings and underscore the role of the C-terminal hub of FtsZ for their correct placement.

## Introduction

The correct placement of the septum contributes to produce two equal daughter cells in the process of *Escherichia coli* division. Both nucleoid occlusion (NO) and the Min system determine the placement of the septum at mid-cell. Their target is the cytoplasmic FtsZ protein, a tubulin homolog that organizes into a ring-like structure (the FtsZ-ring) at mid-cell and functions as a scaffold for the recruitment of at least nine other essential cell division proteins that finally form the bacterial divisome [1], [2], [3], [4]. In *E. coli*, NO is effected by the nucleoid-associated protein SlmA and specific SlmA-binding sequences (SBSs) distributed along the nucleoid [5], [6], [7]. The SlmA protein forms dimers or oligomers that block the higher order assembly of FtsZ protofilaments in the space surrounding the nucleoid, and this activity is significantly enhanced by its previous binding to an SBS [6], [8]. As a result, NO prevents the formation of the FtsZ-ring in the vicinity of nucleoids, thus avoiding the accidental cleavage of unsegregated nucleoids by the constricting division-ring (anti-guillotine effect) [9], [10], [11].

The Min system comprises MinC, MinD and MinE [12], with MinC being a weak cell division inhibitor that blocks FtsZ polymerization [13]. Upon ATP binding, MinD, a peripheral membrane ATPase, interacts with the cytoplasmic membrane through a carboxyl-terminal (C-terminal) amphipathic helix [14], [15]. This interaction of MinD with the membrane increases its affinity for cytoplasmic MinC, leading to the formation of a membrane-bound MinCD complex that is a more potent inhibitor of FtsZ-ring formation [16].

The septum site-selection activity of Min depends on the oscillatory behaviour of its components. A pole-to-pole oscillation of the MinCD pair [17], [18] is enforced by MinE, the third component of the system. Upon binding to MinCD, MinE redistributes the MinCD complex to regions of the cell in which the MinE concentration is lowest, resulting in concentration of MinCD at the opposite cell pole, where the process repeats. As a result, the FtsZ-ring forms at mid-cell, where the concentration of the MinCD inhibitor is statistically the lowest [19].

The effects of NO and Min have been studied in mutants defective in proteins from either system, but it has not yet been possible to study the activity of Min in the complete absence of NO elements, as cells with mutated SlmA or SBSs still contain the nucleoid. To dissect the behaviour of NO and Min, we have designed an experimental system using nucleoid free cells (maxicells), in which NO is completely absent but contains a functional Min. In *E. coli* maxicells, the nucleoid becomes degraded because of irreversible damage induced by ultraviolet (UV) light, but cells remain proficient at transcribing and translating genes encoded in plasmids that, due to their small size, escape UV damage [20].

FtsZ contains a C-terminal domain responsible for its interaction with MinC. In maxicells, we have studied the distribution of the cytoplasmic FtsZ^+^ and a fluorescently tagged variant, and compared that with the distribution of FtsZ*, a mutant lacking the MinC-interacting domain. In addition, the distribution of FtsZ*-VM, a variant artificially targeted to the cytoplasmic membrane, has been studied [21]. Our results in maxicells provide clues to how the distribution of the FtsZ protein is affected by the oscillation of the Min system in the total absence of NO.

## Materials and Methods

### Strains and media

The Escherichia coli strains used in this study were CSR603 (F^−^, ara-14, argE3, galK2, gyrA98 (nalA98), leuB6, lacY1, mtl-1, proA2, phr-1, recA1, rpsL31, supE44, thi-1, tsx-33, thr-1, uvrA6, xyl-5, l^−^) for production of maxicells [20], DH5α (F*−, endA1, glnV44, thi-1, recA1, relA1, gyrA96, deoR, nupG, Φ80dlacZΔM15, Δ(lacZYA-argF)U169, hsdR17(rK− mK+), λ−)* [22] for cloning procedures, VIP2 (F^−^, *λ−, rph-1*, ftsZ::kan/pLAR10 ftsZ^+^, rep(ts), cam^R^) [23] as an ftsZ thermonull strain used to deplete FtsZ at 42 °C, and MC4100 (F*−, [araD139]B/r, Δ(argF-lac)169, λ−,e14-, flhD5301, Δ(fruK-yeiR)725(fruA25), relA1, rpsL150(strR), rbsR22, Δ(fimB-fimE)632(::IS1), deoC1)* [24] as template for polymerase chain reaction (PCR) to amplify *ftsZ*. Bacterial cells were cultured in Luria Bertani broth (LB) or LB agar supplemented with antibiotics when required at a concentration of 100 μg ml^−1^ of ampicillin, 50 μg ml^−1^ of kanamycin or 50 μg ml^−1^ of chloramphenicol [25].

### Plasmids

Plasmids and oligonucleotide primers used are listed in Table 1 and Table S1, respectively. If not mentioned otherwise *Pfu* polymerase (Stratagene) and a primer annealing temperature of 55 °C was used for standard PCR [25]. The gene coding for the FtsZ*-VM (FtsZΔ366-383-Venus-mts) protein was transferred by PCR from pET-FtsZ-mts [21] into pGEM-T vector using primers MP17 and MP18, and subcloned into pTrc99A producing pPZV42. The genes used for the YFP-FtsZ (full length FtsZ) fusion protein were transferred by PCR from plasmid pDSW208-Z338-YFP (*yfp*) and *E. coli* strain MC4100 (*ftsZ*), using primers 1491 and 1478, and 1455 and 1469 respectively, into pKG110 plasmid producing pPZV65. The construct was transferred by PCR using primers 1503 and 1469 into pDSW210 producing pPZV110. The gene fusion encoding for YFP-FtsZ* (YFP-FtsZΔ366-383) was transferred by PCR from pPZV110 using primers MP43 and MP42 into pDSW210 producing pPZV137. The *ftsZ^+^* gene of plasmid pPZV110 was transferred by PCR using the primers MP44 and MP45 into pDSW210 producing pPZV138.

**Table 1:** Plasmids used in this work.

Name	Backbone	Characteristics	Reference
pDR113	pMLB1115	P_Lac_::*minE-gfpmut2*	[27]
pDR119	pMLB1113	P_Lac_::*gfpmut2-minD*	[18]
pDSW208-Z338-YFP	pDSW208	*ftsZ'::eyfp::'ftsZ* (YFP inserted between the 338 and 339 FtsZ residues)	[49]
pDSW208/210	pTrc99A	AmpR, Promoter down mutations in -35 and -10 of pTrc99A	[38]
pET-FtsZ-mts	pET11-b	P_T7_::*ft*sZ(Δ1099-1152 bp)::*venus*::*minD*(Δ1-762 bp)	[21]
pKG110	pLC112	CamR, P_nah_	[50]
pPZV42	pTrc99A	P_Trc_:: *ft*s*Z*(Δ1099-1152 bp)::*venus*::*minD*(Δ1-762 bp)	This work
pPZV46	pET28-a	P_T7_::*his_6_-minE*	This work
pPZV65	pKG110	*yfp::ftsZ*	This work
pPZV110	pDSW210	*yfp::ftsZ*	This work
pPZV137	pDSW210	*yfp*::*ft*s*Z*(Δ1099-1152 bp)	This work
pPZV138	pDSW210	*ftsZ*	This work
pTrc99A	N/A	P_Trc_, amp^R^	[37]

### *E. coli* maxicells

Maxicells were prepared as reported previously [26]. Maxicell preparations are heterogeneous in terms of the number of cells that induced chromosome degradation and escaped lysis by D-cycloserine. Cells that do convert into maxicells maintain intact plasmids; as a result, they can produce sufficient quantities of fluorescently labelled proteins encoded by these plasmids. Not all maxicells that contained either pDR113 (MinE-GFP) or pDR119 (GFP-MinD) and that showed GFP fluorescence exhibited Min oscillations. The percentage of the cells that showed fluorescence and the percentage displaying fluorescent oscillations from pole-to-pole was 22% and 14% respectively for cells with pDR113, and 40% and 21% for cells with pDR119. For maxicells carrying pPZV110, 33% showed YFP fluorescence and 27% of these cells localized YFP-FtsZ at midcell. In the case of pPZV137, 27% of maxicells showed YFP fluorescence and 22% of these cells contained YFP-FtsZ* polymers localized throughout the entire cell.

### Production of plasmid-encoded protein in maxicells

To produce GFP-MinD or MinE-GFP, *E. coli* CSR603 cells carrying plasmid pDR119 or pDR113, respectively, were grown in LB supplemented with ampicillin to 1×10^8^ cells per ml in a shaker at 37 °C. At this point, cells were either converted into maxicells or processed as non UV-irradiated *wild-type* controls. The production of either GFP-MinD or MinE-GFP in maxicells was induced during the maxicell procedure [26] by the addition of 25 μM IPTG (Isopropyl β-D-1-thiogalactopyranoside) 3 hours after UV irradiation together with the addition of D-cycloserine, and incubated for 16 hours. This amount of IPTG is sufficient for the induction of expression of the GFP fusions, but not toxic for cell growth [27]. Exposures of the fluorescence images were adjusted to correct for photobleaching of the GFP fluorescence. For the production of FtsZ^+^, YFP-FtsZ, YFP-FtsZ* or FtsZ*-VM, *E. coli* CSR603 carrying either pPZV138, pPZV110, pPZV137 or pPZV42, respectively, were grown in LB supplemented with ampicillin in a shaker at 37 °C until they reached 1×10^8^ cells ml^−1^. The production of these proteins during the maxicell procedure was induced by the addition of 1 mM IPTG. Production of FtsZ*-VM during the maxicell procedure was induced by the addition of 0.5 mM IPTG followed by incubation for 16 hours. We used different IPTG concentrations to account for the different plasmid backbones (Table 1).

### Production of plasmid-encoded proteins in *E. coli* VIP2

To produce FtsZ^+^, YFP-FtsZ or YFP-FtsZ* in *E. coli* VIP2, strains carrying plasmids pPZV138, pPZV110 or pPZV137 respectively, were grown overnight (ON) in LB supplemented with kanamycin, chloramphenicol and ampicillin in a shaker at 30 °C. The next day, a 1∶100 dilution of the ON culture was inoculated in fresh pre-warmed LB supplemented with kanamycin, chloramphenicol and ampicillin and grown in a shaker at 30°C to an optical density OD_600_ of 0.3. The cell culture was shifted to 42 °C for 180 minutes to deplete the cells of endogenous FtsZ [28]; the production of FtsZ^+^, YFP-FtsZ or YFP-FtsZ* was induced by the addition of 15 μM IPTG for additional 150 min. The production of FtsZ*-VM was induced by the addition of 0.5 mM IPTG. Cells were prepared for phase-contrast and fluorescence microscopy without fixation.

### Complementation of filamentous VIP2 phenotype on plates

Plasmid-encoded proteins FtsZ^+^, YFP-FtsZ and YFP-FtsZ* were tested for functionality in strain VIP2 [23] at 42 °C. Cultures of VIP2 carrying either pPZV137 (YFP-FtsZ*), pPZV110 (YFP-FtsZ), pPZV138 (FtsZ^+^) or the empty vector control (pDSW210) were grown ON at 30 °C and spotted in a 10-fold dilution series on LB plates supplemented with kanamycin, chloramphenicol, ampicillin and 0, 5, 15 or 100 μM IPTG; these were then incubated ON at 30 °C or 42 °C.

### Microscopy

Phase-contrast and fluorescence microscopy was performed as described in Pazos *et al.* [26] using an Olympus U-MNIBA2 filter (excitation filter: 470–490 nm; emission filter: 515–550 nm; dichroic filter: 505 nm) to visualize the GFP and YFP fluorescence [29]. Time-lapse fluorescence images of maxicells producing YFP-FtsZ* were recorded with a Hamamatsu ORCA 3-CCD camera. Cell lengths were measured with the Object-Image plug-in [30] of the ImageJ software package [31]. The Huygens Professional software package with a correction factor of 0.42 (calculated from the measurement of control spherical particles) was used to deconvolve fluorescence microscope images. Immunolabeling of bacterial cells was performed as described [32], using purified FtsZ (MVC1), FtsA (MVM1) or ZipA (MVC1) antibody as primary antibody and Alexa Fluor-594 labelled goat anti-rabbit IgG (Invitrogen) as secondary antibody. Line profiles of fluorescent signals were analysed using ImageJ [31].

### General methods

SDS-PAGE [33] and western blot analysis [34] were performed according to standard procedures. For SDS-PAGE, bacterial culture pellets were resuspended in SDS-PAGE sample buffer, boiled for 5 minutes at 95 °C and the cell equivalent of an *A*_600_ of 0.1 was loaded per lane. Rabbit antisera or purified antibodies of anti-FtsZ (MVC2), anti-FtsA (MVM1), anti-SlmA, anti-MinC, anti-MinD, anti-MinE (MVZ1) (supplemental material) and anti-ZipA (MVC1) were used for specific protein detection. Horseradish peroxidase-coupled protein A (Bio-Rad), BM chemiluminescence blotting substrate (POD, Roche Molecular Biochemicals) and either BioRAD ChemiDoc XRS+ Imaging System or Kodak Biomax XAR film were used for developing the luminescence signals on PVDF (Millipore) or Nitrocellulose (Roche) membranes. Quantification of the immunoblotted protein levels was performed using ImageJ.

## Results

### Absence of the nucleoid and SlmA in maxicells

**Figure 1:**
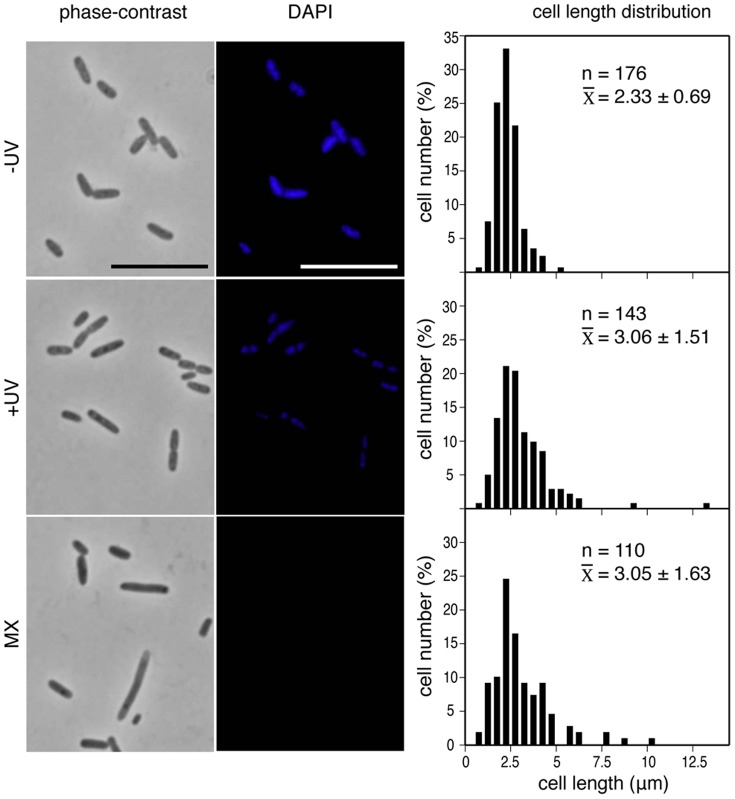
Morphological parameters of *E. coli* maxicells. Maxicell samples were withdrawn at different stages during the maxicell production procedure. Namely, non UV-irradiated cells (−UV); cells at 3 hours after UV irradiation (+UV); and maxicells (MX) corresponding to UV irradiated cells incubated for 3 hours and then supplemented with D-cycloserine and incubated for additional 16 hours. See text for details. Panels at the left show phase contrast images of representative fields. The panels at the center show DAPI fluorescence images of the same fields. Scale bars  = 10 μm. The panels at the right show cell length distributions for each sample measured as described in the text. The number of cells measured, n, the mean cell length, x, and the standard deviation are indicated.

**Figure 2:**
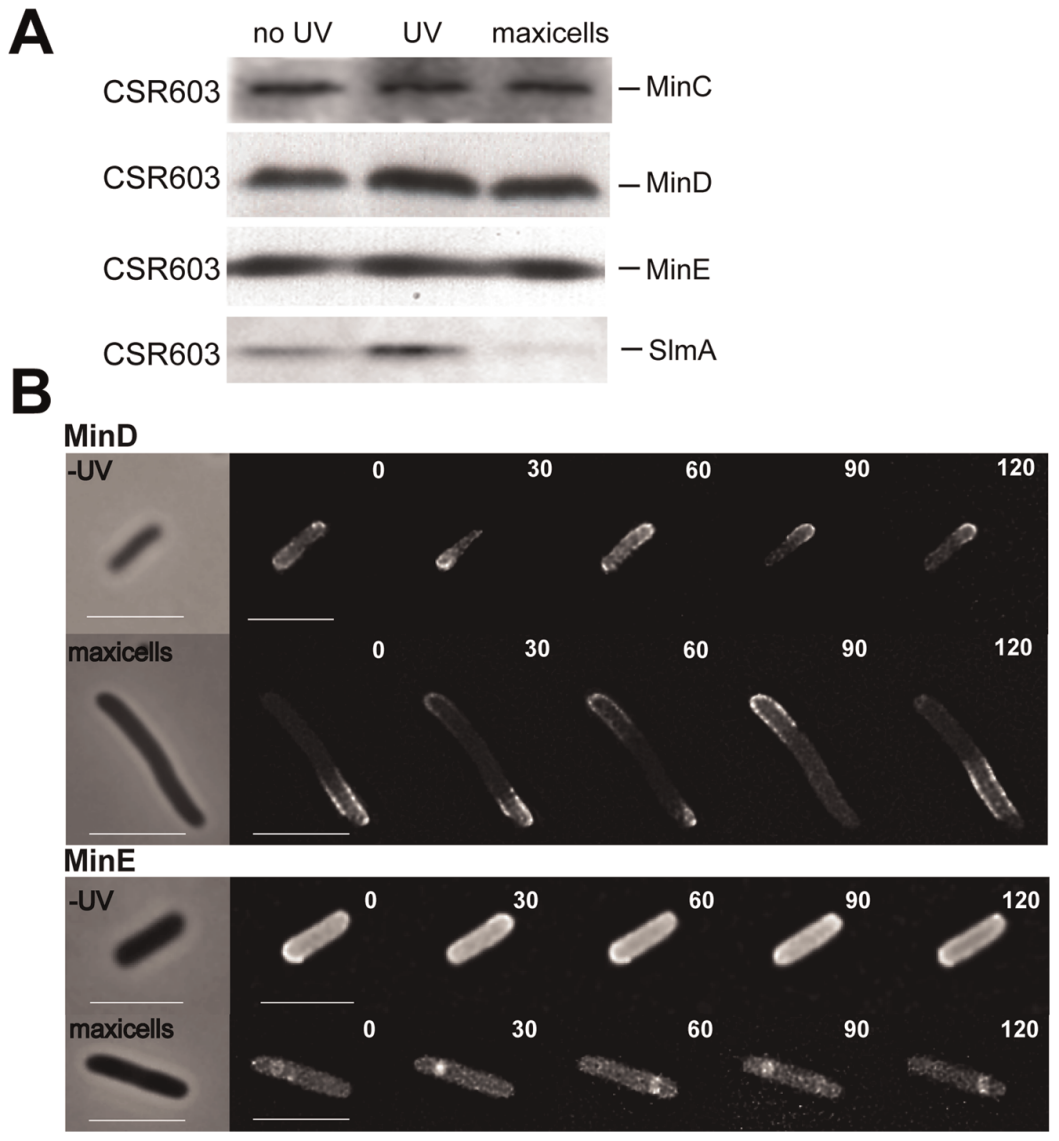
Levels of septum site-selection proteins and oscillation of MinD and MinE in the presence and absence of the bacterial nucleoid. (A) Samples of *E. coli* CSR603 withdrawn at the stages described in Figure 1 show the amounts of SlmA, MinC, MinD and MinE measured by immunoblotting with specific antibodies (see Material and Methods). Cells corresponding to an *A*_600_ of 0.1 were loaded per lane. (B) Phase-contrast and deconvolved GFP-fluorescence microscopy images of non-UV irradiated CSR603 cells (−UV) and CSR603 maxicells carrying plasmid producing either GFP-MinD or MinE-GFP were captured at the times (seconds) indicated in the different frames. A phase contrast image, taken at time zero, has been included for each case (left hand side frames). Scale bars  = 5 μm.

Maxicells are bacterial cells lacking chromosomes [20], so we tested our maxicell preparations for the presence of chromosomal DNA and the nucleoid associated protein SlmA, which binds simultaneously to the chromosomal DNA and FtsZ [6], [8]. After low-dose UV irradiation, CSR603 cells were treated as described in *Materials and Methods* to produce maxicells [20]. DAPI staining of the maxicell preparation indicated that most chromosomal DNA was degraded, as the fluorescence intensity in cells decreased at 3 hours after UV-irradiation and was barely detectable in the final maxicell sample (Figure 1). Cell morphology and cell length distributions show that the mean cell length of the maxicell population was not significantly affected throughout the preparation procedure, although the presence of a few longer cells, relative to the unirradiated population, could be detected (Figure 1). The levels of SlmA, the effector protein of NO in *E. coli,* increased at 3 hours after irradiation (195%) when compared to levels present in non-UV-irradiated cells (100%), but SlmA was not detectable in maxicells (25%) (Figure 2A). We do not know if the degradation of SlmA in maxicells reflects a natural rapid turnover or differential stability of the protein when not bound to DNA. Why the levels of SlmA increase after UV-irradiation is not known, although it could be explained if the *slmA* gene formed part of a RecA-independent repair pathway. Nevertheless, our results, together with the degradation of the bacterial nucleoid, further define maxicells as cells in which the NO septum selection mechanism is absent.

### MinD and MinE oscillate in maxicells

Measurements of the levels of MinC, MinD and MinE indicate that the concentrations of these proteins involved in the Min septum site selection system remain unchanged in maxicells (Figure 2A). To find if the Min system is functional in maxicells, we asked whether normal pole-to-pole oscillation of the Min proteins could be observed in the absence of the bacterial nucleoid. In non-UV irradiated cells, MinD and MinE are required for oscillation [27]. MinC, on the other hand, is defined as a passenger protein, as it is not itself attached to the membrane and oscillates passively due to its high affinity binding to MinD [35]. To monitor the position of MinD or MinE in maxicells, we produced GFP-MinD or MinE-GFP under the control of the IPTG-inducible *lac* promoter (from pDR119 and pDR113 respectively). The fluorescence signals from either GFP-MinD or MinE-GFP exhibited pole-to-pole oscillation in maxicells with a period of 40–60 seconds, comparable to the value found for the oscillation of fluorescently tagged MinD or MinE in non-UV irradiated growing cells (Figure 2B and [18]). This indicates that Min oscillation in maxicells occurs in the absence of the nucleoid, and that oscillations independent of NO parallel the oscillations exhibited in non UV-irradiated cells. The deconvolved fluorescence microscopy images show that GFP-MinD appeared as regularly-spaced spots associated with the membrane that could be interpreted as forming a spiral structure along the longitudinal cell axis, and that MinE-GFP oscillates as a sharp band or ring (Figure 2B). The normal oscillation of MinD and MinE in maxicells also indicates that ATP is available at sufficient levels to drive MinD dimerization and association with the cytoplasmic membrane, the two requisites for the binding of MinC [36]. We infer that the oscillation of MinDE should also drive the displacement of the MinD-attached MinC to form a functional Min system, as the levels of MinC remain unchanged in maxicells (Figure 2A).

### Biological functionality of YFP-FtsZ and YFP-FtsZ*

**Figure 3:**
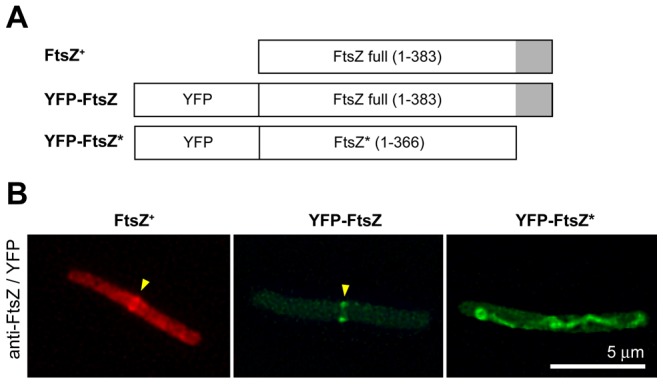
Localization of FtsZ variants in maxicells. (A) Schematic representation of the FtsZ proteins carried by each plasmid used in panel B. The grey shaded square represents the 17 amino acid residues of the carboxyl-terminal domain of FtsZ. This domain is not present in YFP-FtsZ*. (B) Maxicells obtained from CSR603 cells containing plasmids pPZV138 (FtsZ^+^), pPZV110 (YFP-FtsZ) or pPZV137 (YFP-FtsZ*) were observed after 16 hours induction of the plasmid encoded genes by 1 mM IPTG. The FtsZ protein was immunostained with Alexa Fluor-594 labelled MCV2 purified antibody against FtsZ. YFP-FtsZ and YFP-FtsZ* were visualized by YFP fluorescence. See also the recorded YFP fluorescence images of YFP-FtsZ* in Movie S1.

We constructed three plasmids encoding FtsZ^+^, YFP-FtsZ or YFP-FtsZ*. YFP-FtsZ is a fusion of the fluorescent protein YFP to the amino terminal (N-terminal) end of FtsZ. YFP-FtsZ* is similar to YFP-FtsZ except that it lacks the FtsZ C-terminal hub, *i.e.* the residues 367-383 at the C-terminal end (Figure 3A). The biological functionalities of FtsZ^+^, YFP-FtsZ and YFP-FtsZ* were tested by their ability to support the growth of VIP2, a *ftsZ* thermonull strain in which the synthesis of FtsZ is abolished at 42 °C. Although VIP2 has an insertional inactivation of the chromosomal *ftsZ* gene, it can grow normally at 30 °C because it bears an extra copy of *ftsZ^+^* on the replication-thermosensitive plasmid pLAR10 [23]. The expression of this *ftsZ* ^+^ is controlled by the native transcriptional and translational controls present in the cloned fragment. Upon transfer to 42 °C, a filamentous phenotype is produced because of depletion of FtsZ following the dilution of the thermosensitive plasmid.

We first found that even in the absence of IPTG, expression from the strong Trc promoter [37] yields amounts of FtsZ that inhibit cell division and growth (data not shown). To circumvent this problem, we used a weakened Trc promoter derivative in plasmid pDSW210 that allows lower expression of cloned genes [38]. Plasmids pDSW210 (empty control), pPZV138 (FtsZ^+^), pPZV110 (YFP-FtsZ) or pPZV137 (YFP-FtsZ*) were transformed into VIP2. Transformants were then grown at 30 °C and cultures transferred to 42 °C to measure viability on solid medium and cell length in liquid cultures. This weakened Trc promoter is still leaky, allowing the expression of *ftsZ^+^* at levels sufficient to support the growth of VIP2 even in the absence of inducer.

**Figure 4:**
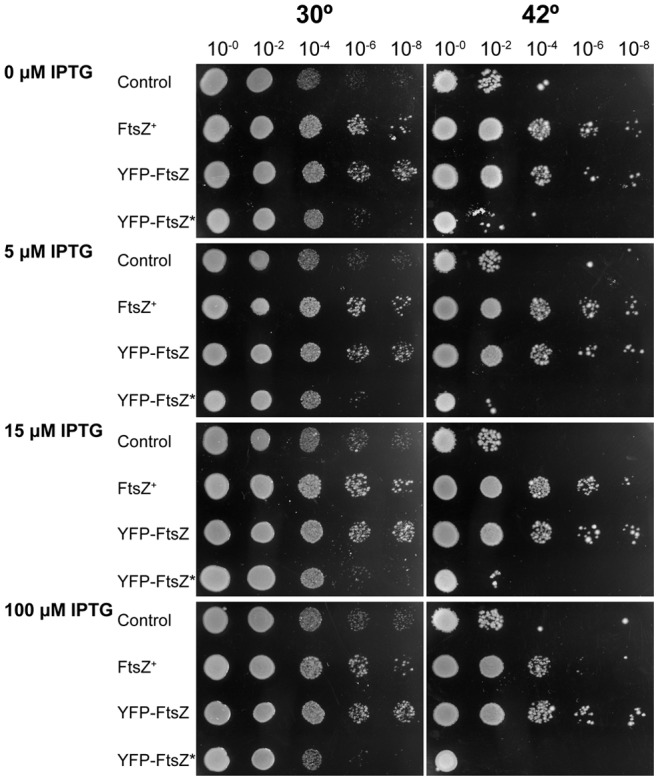
Complementation of the growth defect of the thermonull (VIP2) *E. coli ftsZ* strain. 10-fold serial dilutions of spotted VIP2 strain carrying plasmid pDSW210 (control), pPZV138 (FtsZ^+^), pPZV110 (YFP-FtsZ) or pPZV137 (YFP-FtsZ*) grown overnight at 30 °C (left) and 42 °C (right). The concentrations of IPTG on the LB plates are indicated in the figure.

The results show that plasmids encoding FtsZ^+^ or YFP-FtsZ were able to restore growth and viability of VIP2 at 42 °C (Figure 4). In the presence of 15 μM IPTG, cells containing pPZV110 (YFP-FtsZ) were slightly longer (4.4 μm) than those transformed with pPZV138 (FtsZ^+^) (3.0 μm) (Figure S1). On the other hand those cells containing pPZV137 (YFP-FtsZ*) or the control plasmid failed, as expected, to restore growth and viability or to allow cell length to return to normal values (Figures 4, S1 and S2). We conclude that YFP-FtsZ, although able to complement VIP2 at 42 °C, was not functional to the same extent as FtsZ^+^ as it drove septation at longer than wild-type cell lengths. In contrast, YFP-FtsZ*, missing the FtsZ central hub, was not functional at all. High amounts of YFP-FtsZ*, for example produced after 100 μM of IPTG induction, were toxic (Figure 4). The toxicity was detectable at 42 °C, reducing the already low viability by less than an additional order of magnitude, and it was more severe at 30 °C (two orders of magnitude reduction). These results agree with the dominant negative effect of a protein containing the N-terminal domain of FtsZ in the absence of its functional C-terminal domain [39], [40]. These results suggest that at 30 °C, YFP-FtsZ* may interfere with the function of the FtsZ^+^ encoded in the chromosome.

### The Min system alone directs the positioning of FtsZ in maxicells

The absence of NO and the presence of a functional Min system in maxicells prompted us to use maxicells to study the placement of FtsZ, their divisome target. We have previously shown that FtsZ is extensively degraded by the ClpXP system in maxicells [26]. As a consequence, plasmid-encoded FtsZ can then be produced *de novo* and its localization can be monitored in the absence of the bulk of the chromosomally encoded protein. We induced the production of FtsZ^+^, YFP-FtsZ or YFP-FtsZ* in maxicells containing suitable plasmids by adding 1 mM IPTG 3 hours after UV-irradiation (Figure S3). The percentage of cells showing a YFP fluorescent signal within a maxicell population was nearly 30% of the total, suggesting that the remainder of the maxicells lacked intact plasmid DNA, either as a result of rare plasmid loss in the original population or DNA degradation after irradiation damage.

We localized FtsZ^+^ or YFP-FtsZ by immunofluorescence in fixed cells. The results show that the number of cells containing sharp FtsZ rings at midcell was negligible when maxicells contained a control plasmid and increased to over 5% when a plasmid containing *ftsZ^+^* was present (Figure 3B and Table 2). A similar increase in the percentage of cells showing sharp rings at midcell was observed by measuring immunofluorescence when the plasmid produced YFP-FtsZ, suggesting that the presence of the YFP tag did not produce major alterations in the localization of the fusion protein.

**Table 2:** FtsZ localization in maxicells.

	Immunofluorescence	YFP fluorescence			
Protein	% midcell rings (n)	% fluorescent signal (n)	% midcell sharp rings	% dispersed multiple rings	% unstructured polymers
None	0.0 (176)	NA	NA	NA	NA
FtsZ^+^	5.2 (401)	NA	NA	NA	NA
YFP-FtsZ	5.8 (330)	33.1 (139)	27.3	5.8	0.0
YFP-FtsZ*	0.0 (152)	27.0 (102)	0.0	0.0	21.6

%, percentage of cells in each category.

*n*, total number of cells counted.

We also examined live cells with YFP-FtsZ, and found that a high percentage exhibited fluorescent rings at mid-cell (Figure 3B and Table 2). Immunofluorescent detection of FtsZ had a high fluorescence background, presumably due to antibody non-specificity and residual amounts of proteins contained in maxicells. This may be among the reasons why a lower number of sharp rings were detected using immunofluorescence compared with the number from YFP-FtsZ fluorescence in which no background is observed.

Both ZipA and FtsA tether FtsZ^+^ to the membrane in normal growing *E. coli* cells. Maxicells retain normal levels of these two proteins and both remain in close proximity to the cytoplasmic membrane [41]. Neither protein showed preferential localization along the cell axis in the absence of plasmids encoding FtsZ (Table 3). We then determined the localization of ZipA and FtsA in maxicells when FtsZ^+^ or YFP-FtsZ were produced. Interestingly, production of either FtsZ^+^ or YFP-FtsZ resulted in the midcell localization of ZipA but not FtsA (Figure S4). From all these results, we suggest that the inhibitory action of MinC and the oscillatory behaviour of MinCDE are sufficient for the placement of both FtsZ^+^ and YFP-FtsZ at midcell in the complete absence of NO in maxicells.

**Table 3:** Immunolocalization of FtsA and ZipA in maxicells overproducing different FtsZ variants.

	% midcell ZipA (n)	% midcell FtsA (n)
**Control**	0.6 (170)	0 (115)
**FtsZ+**	15 (100)	0 (209)
**YFP-FtsZ**	3.1 (513)	0 (218)
YFP-FtsZ*	0 (159)	0 (187)

%, percentage of cells in each category.

*n*, total number of cells counted.

### FtsZ localization at midcell depends on the presence of its carboxyl-terminal domain

**Figure 5:**
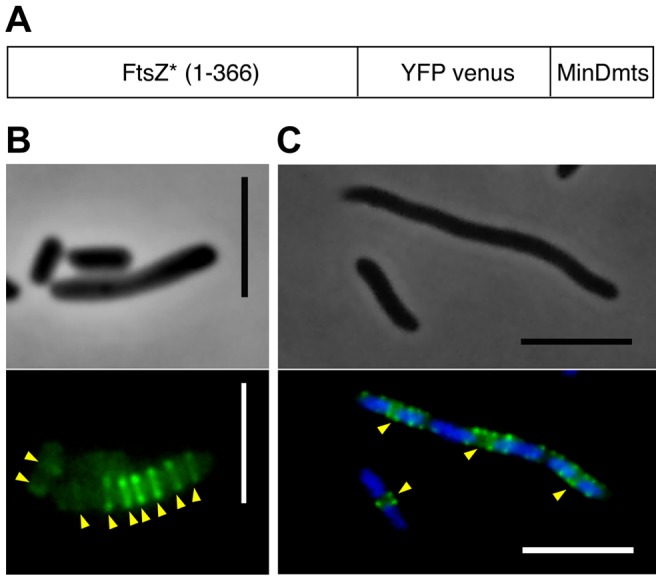
Localization of FtsZ*-VM in maxicells and in FtsZ-depleted VIP2 cells. (A) Schematic representation of the FtsZ variant carried by plasmid pPZV42. The 17 amino acid residues of the carboxyl-terminus domain of FtsZ are not present in FtsZ*-VM. (B) Maxicells obtained from CSR603 cells containing plasmid pPZV42 (FtsZ*-VM) were observed after 16 hrs induction of the plasmid encoded genes by 0.5 mM IPTG. (C) VIP2 cells harbouring plasmid pPZV42 (FtsZ*-VM) were grown as indicated in *Materials and Methods*. The fluorescence signal of the Venus fusions in B and C was observed as indicated in *Materials and Methods*. Arrowheads mark the position of the FtsZ-rings. Scale bars  = 5 μm.

The C-terminal domain of FtsZ is a central hub that interacts with different proteins during divisome assembly, among them ZipA [42], FtsA [43] and the FtsZ-ring inhibitor MinC [13]. In the absence of NO, the MinCDE system should not be able to restrict the cellular spreading of YFP-FtsZ*, lacking the C-terminal domain of FtsZ. As expected, this protein localizes as a cytosolic polymer along the length of the maxicells (Figure 3B and Movie S1). Osawa *et al.* constructed an FtsZ*-VM mutant similar to YFP-FtsZ* that lacks the C-terminal domain and was fused to the Venus variant of YFP. In addition, FtsZ*-VM contains the membrane targeting sequence of MinD (MinD_mts_) (Figure 5A) to provide an anchoring to the membrane independent of FtsA and ZipA [21]. In maxicells, the membrane-attached FtsZ*-VM localizes as multiple rings along the length of the cell (Figure 5B). When reconstituted in the presence of tubular lipid vesicles *in vitro*, this protein was also distributed as FtsZ-rings [21]. We conclude that the absence of the C-terminal domain in FtsZ*-VM and in YFP-FtsZ* leads to the misplacement of FtsZ-rings in maxicells.

### The role of NO in positioning Min-insensitive FtsZ variants at midcell

**Figure 6:**
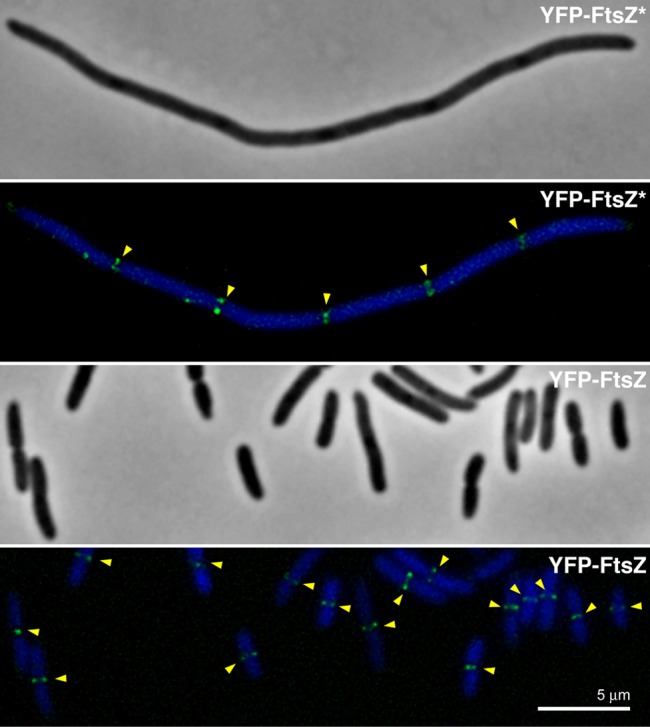
Localization of YFP-FtsZ and YFP-FtsZ* in FtsZ-depleted VIP2 cells. Phase-contrast and fluorescence merged images of DAPI staining and YFP-fluorescence signal are shown for VIP2 cells producing YFP-FtsZ or YFP-FtsZ* at 42 °C after FtsZ depletion. Each sample was stained with DAPI to evidence bacterial nucleoids. Arrowheads mark the position of the FtsZ-rings.

YFP-FtsZ* and FtsZ*-VM failed to localise at specific places in the absence of NO in maxicells, but these fusion proteins cannot interact normally with MinC because they lack the FtsZ central hub. We wanted to investigate how these FtsZ derivatives localised in cells with both active NO and Min systems. For this purpose we expressed plasmids encoding either YFP-FtsZ*, FtsZ*-VM, YFP-FtsZ or FtsZ^+^ in strain VIP2, in which native FtsZ can be depleted to levels below 20% at 42 °C. Immunofluorescence (for FtsZ^+^) and fluorescence microscopy images (for the rest) of these FtsZ variants showed that they all localize between the segregated nucleoids at potential septation sites (Figure S1 and Figure 6). FtsZ^+^ and YFP-FtsZ formed single FtsZ-rings, whereas FtsZ*-VM assembled as multiple rings or perhaps short spirals sandwiched between the segregated nucleoids (Figure 5C and S5). Although YFP-FtsZ* lacks the C-terminal region that interacts with the FtsZ membrane-anchoring ZipA or FtsA proto-ring counterparts, it nonetheless localizes as single rings between nucleoids. These results indicate that NO is able to position FtsZ proteins, including the Min-insensitive YFP-FtsZ* and FtsZ*-VM, at mid-cell or potential division sites. Irrespective of their correct placement, YFP-FtsZ* and FtsZ*-VM are not able to drive septation. In contrast, YFP-FtsZ supports septation in VIP2 at the restrictive temperature almost as efficiently as FtsZ^+^ as shown above (Figure 4 and S1).

## Discussion

Placement of a septum at midcell is important for bacteria to divide into approximately two equal halves and to avoid accidental strangling of the nucleoid before it is fully replicated and segregated. It is not surprising that at least two systems, Min and NO, have been found to accurately control the placement of FtsZ, the initial protein assembling into the proto-ring. Under normal circumstances, the combined action of the two systems prevents septation at the poles and premature septation at midcell, as well as concentrates FtsZ to form a ring at midcell after the nucleoids are separated.

In this work we have first dissected NO from Min using maxicells, in which NO is fully absent while Min is functional. We found that the Min system on its own can position FtsZ correctly at midcell. To separate the action of NO from Min we constructed FtsZ derivatives, YFP-FtsZ* and FtsZ*-VM, lacking the central hub of FtsZ, its C-terminal domain responsible for the interaction with MinC and other proteins. To avoid interference from their possible association with FtsZ^+^, these proteins have been produced under conditions in which the levels of the wild type protein are insufficient for localization and the number of FtsZ^+^-rings is negligible [23]. In these conditions we found that NO is sufficient to place YFP-FtsZ* or FtsZ*-VM at nucleoid-free spaces and exclude them from regions overlapping nucleoids. The presence of two apparently redundant systems for septum placement can be justified if Min simply prevented assembly of a FtsZ-ring at the cell poles, resulting in a rather broad area for FtsZ to assemble, whereas NO prevented guillotining of the nucleoid by the septum independent of cell geometric cues. It is only when Min and NO work together that septation is preferentially targeted to midcell and a nucleoid-free regions.

Under normal circumstances, assembly of FtsZ into a ring requires its anchoring to the cytoplasmic membrane through its binding partners FtsA and ZipA. The stability of these two proteins is not significantly compromised in maxicells [41]. We observed that FtsZ^+^ and YFP-FtsZ localize as FtsZ-rings at midcell in maxicells (Figure 3). These two proteins are proficient at localizing ZipA at midcell in maxicells but they fail to similarly localize FtsA. ZipA and FtsA, although both are required for the early assembly of the proto-ring, may nevertheless act asynchronously in the attachment of FtsZ polymers to the membrane. In turn, YFP-FtsZ*, the variant lacking the central hub, forms long unstructured dynamic protein filaments in the cytoplasm (Figures 3 and Movie S1).

Further information regarding the effect of membrane anchoring on the placement of the FtsZ-ring can be derived from the behaviour of FtsZ*-VM, a Min-insensitive protein that is artificially targeted to the membrane by the short amphipathic helix from MinD (MinD_mts_). In the absence of NO, this protein forms many FtsZ-rings that are randomly distributed from pole to pole along the length of the maxicells instead of forming filaments in the cytoplasm (Figure 5). This emphasizes the need for a membrane anchor provided naturally by ZipA and FtsA or artificially by MinD_mts_, to assemble FtsZ into rings. These FtsZ*-VM rings remain separate as multiple discrete bands when bound to the membrane of the maxicell, suggesting that the membrane-bound FtsZ-rings produce an exclusion zone in which the lateral interaction between adjoining rings is prevented. The existence of a molecular mechanism responsible for this exclusion effect may be postulated and has been observed as well in FtsZ-rings attached to the membrane through ZipA [44]. Contrary to this and to our observation in maxicells, in which the envelope structure is also conserved, rings of FtsZ*-VM have been shown to coalesce when reconstituted in tubular liposomes [21]. This may due to the absence of site selection components in the liposome, or perhaps some general aspect of normal cell envelope structure helps to establish the putative exclusion effect.

The existence of additional mechanisms to direct septum positioning at midcell independently of both Min and NO in *E. coli* has been suggested by Mannik *et al.* [45], Cambridge *et al.* [46] and Potluri *et al.* [47] and in *Bacillus subtilis* by Rodrigues *et al*. [48]. The molecular basis underlying these possible mechanisms has not been precisely determined. Our results suggest that they might operate on the C-terminal hub of FtsZ, as the Min insensitive YFP-FtsZ* and FtsZ*-VM fail to localise in the absence of NO.

## Supporting Information

Figure S1 **Immunolocalization of the plasmid-encoded FtsZ variants in FtsZ-depleted VIP2 cells.** Cultures of VIP2 cells harbouring plasmids pPZV120 (control), pPZV110 (YFP-FtsZ), pPZV137 (YFP-FtsZ*) or pPZV138 (FtsZ^+^) growing exponentially at 30 °C were shifted to 42 °C and incubated for 180 min to deplete the amount of FtsZ^+^. Production of each plasmid-encoded FtsZ variant was then achieved by addition of IPTG (15 μM) during 150 min. Phase contrast and fluorescence merged micrographs of DAPI staining and FtsZ immunolocalization signal are shown for each strain. Mean cell length ± standart deviation (μm) and number of cells measured (n) are indicated in each case.
(TIF)

Figure S2 Production of FtsZ^+^, YFP-FtsZ and YFP-FtsZ* in FtsZ-depleted VIP2 cells. Immunoblot of extracts from VIP2 transformed with pPZV110 (YFP-FtsZ), pPZV120 (empty vector control), pPZV137 (YFP-FtsZ*) or pPZV138 (FtsZ^+^) revealed using MVC2 anti-FtsZ serum. The transformants were grown at 30 °C until they reached balanced exponential growth rate (lane: 0 min). At time 0 they were shifted to 42 °C and incubated for 180 min to deplete the amount of FtsZ^+^ (lane: 180 min). Production of each plasmid-encoded FtsZ variant was then achieved by addition of IPTG (15 μM) during 150 min (lane: +IPTG). The amount of FtsZ in each sample, relative to the amount of FtsZ present at 0 min (100%), is indicated below each lane.
(TIF)

Figure S3 Production of FtsZ^+^, YFP-FtsZ and YFP-FtsZ* in maxicells. Western blot showing the protein level of the FtsZ variants during the maxicell procedure of *E. coli* CSR603 bearing pPZV110 (YFP-FtsZ), pDSW210 (empty vector control), pPZV137 (YFP-FtsZ*) or pPZV138 (FtsZ). (−UV) non-UV irradiated CSR603, (+UV) 3 hours after UV irradiation, (MX) 19 hours after irradiation and (MX+IPTG) 16 hours after addition of IPTG at time-point +UV. Relative amount of FtsZ is indicated below each sample.
(TIF)

Figure S4 **FtsA and ZipA localization in maxicells producing FtsZ variants.** Phase contrast and fluorescence merged micrographs of DAPI staining and FtsA immunolocalization (left) or DAPI staining and ZipA immunolocalization (right). The images correspond to maxicells containing pPZV120 (control), pPZV138 (FtsZ^+^), pPZV110 (YFP-FtsZ) or pPZV137 (YFP-FtsZ*). These samples correspond to those analyzed by Western blot in lane MX+IPTG in Figure S3 (see Figure S3 legend).
(TIF)

Figure S5 Localization of FtsZ*-VM in FtsZ-depleted VIP2 cells. Line profiles of fluorescent signals emanating from the cells shown in Figure 5C. Arbitrary fluorescent units are plotted on the y axis and cell length (in μm) is plotted on the x axis. Grey lines correspond to DAPI staining (nucleoids position) and black lines correspond to FtsZ*-VM fluorescence.
(TIF)

Table S1 **Oligonucleotide primers used in this study.**
(DOC)

Text S1 **Supporting Material and References.**
(DOC)

Movie S1 Localization of YFP-FtsZ* in the absence of NO. YFP fluorescence time-lap images of CSR603 maxicell carrying plasmid producing YFP-FtsZ* were recorded using 2.5 seconds as image exposure time.
(AVI)

## References

[1] Natale P, Pazos M, Vicente M (2013) The Escherichia coli divisome: born to divide. Environ Microbiol 15: 3169–3182. 23962168 10.1111/1462-2920.12227

[2] Rico AI, Krupka M, Vicente M (2013) In the beginning, Escherichia coli assembled the proto-ring: an initial phase of division. J Biol Chem 288: 20830–20836. 23740256 10.1074/jbc.R113.479519 PMC3774354

[3] Egan AJ, Vollmer W (2013) The physiology of bacterial cell division. Ann N Y Acad Sci 1277: 8–28. 23215820 10.1111/j.1749-6632.2012.06818.x

[4] Huang KH, Durand-Heredia J, Janakiraman A (2013) FtsZ ring stability: of bundles, tubules, crosslinks, and curves. J Bacteriol 195: 1859–1868. 23457247 10.1128/JB.02157-12 PMC3624584

[5] Bernhardt TG, de Boer PA (2005) SlmA, a nucleoid-associated, FtsZ binding protein required for blocking septal ring assembly over Chromosomes in E. coli. Mol Cell 18: 555–564. 15916962 10.1016/j.molcel.2005.04.012 PMC4428309

[6] Tonthat NK, Milam SL, Chinnam N, Whitfill T, Margolin W, et al (2013) SlmA forms a higher-order structure on DNA that inhibits cytokinetic Z-ring formation over the nucleoid. Proc Natl Acad Sci U S A 110: 10586–10591. 23754405 10.1073/pnas.1221036110 PMC3696773

[7] Cho H, McManus HR, Dove SL, Bernhardt TG (2011) Nucleoid occlusion factor SlmA is a DNA-activated FtsZ polymerization antagonist. Proc Natl Acad Sci U S A 108: 3773–3778. 21321206 10.1073/pnas.1018674108 PMC3048121

[8] Cho H, Bernhardt TG (2013) Identification of the SlmA Active Site Responsible for Blocking Bacterial Cytokinetic Ring Assembly over the Chromosome. PLoS Genet 9: e1003304. 23459366 10.1371/journal.pgen.1003304 PMC3573117

[9] Jaffé A, Boye E, D'Ari R (1990) Rule governing the division pattern in *Escherichia coli minB* and wild-type filaments. J Bacteriol 172: 3500–3502. 2188963 10.1128/jb.172.6.3500-3502.1990 PMC209166

[10] Sun Q, Margolin W (2004) Effects of perturbing nucleoid structure on nucleoid occlusion-mediated toporegulation of FtsZ ring assembly. J Bacteriol 186: 3951–3959. 15175309 10.1128/JB.186.12.3951-3959.2004 PMC419936

[11] Woldringh CL, Mulder E, Huls PG, Vischer N (1991) Toporegulation of bacterial division according to the nucleoid occlusion model. Res Microbiol 142: 309–320. 1925029 10.1016/0923-2508(91)90046-d

[12] Shih YL, Zheng M (2013) Spatial control of the cell division site by the Min system in Escherichia coli. Environ Microbiol 15: 3229–3239. 23574354 10.1111/1462-2920.12119

[13] Shen B, Lutkenhaus J (2009) The conserved C-terminal tail of FtsZ is required for the septal localization and division inhibitory activity of MinC(C)/MinD. Mol Microbiol 72: 410–424. 19415799 10.1111/j.1365-2958.2009.06651.x PMC2759774

[14] Lackner LL, Raskin DM, de Boer PA (2003) ATP-dependent interactions between Escherichia coli Min proteins and the phospholipid membrane in vitro. J Bacteriol 185: 735–749. 12533449 10.1128/JB.185.3.735-749.2003 PMC142821

[15] Suefuji K, Valluzzi R, RayChaudhuri D (2002) Dynamic assembly of MinD into filament bundles modulated by ATP, phospholipids, and MinE. Proc Natl Acad Sci U S A 99: 16776–16781. 12482939 10.1073/pnas.262671699 PMC139220

[16] de Boer PA, Crossley RE, Rothfield LI (1989) A division inhibitor and a topological specificity factor coded for by the minicell locus determine proper placement of the division septum in *E. coli* . Cell 56: 641–649. 2645057 10.1016/0092-8674(89)90586-2

[17] Hu Z, Lutkenhaus J (2001) Topological regulation of cell division in E. coli. spatiotemporal oscillation of MinD requires stimulation of its ATPase by MinE and phospholipid. Mol Cell 7: 1337–1343. 11430835 10.1016/s1097-2765(01)00273-8

[18] Raskin DM, de Boer PA (1999) Rapid pole-to-pole oscillation of a protein required for directing division to the middle of *Escherichia coli* . Proc Natl Acad Sci U S A 96: 4971–4976. 10220403 10.1073/pnas.96.9.4971 PMC21801

[19] Meinhardt H, de Boer PA (2001) Pattern formation in *Escherichia coli*: a model for the pole-to-pole oscillations of Min proteins and the localization of the division site. Proc Natl Acad Sci U S A 98: 14202–14207. 11734639 10.1073/pnas.251216598 PMC64659

[20] Sancar A, Hack AM, Rupp WD (1979) Simple method for identification of plasmid-coded proteins. J Bacteriol 137: 692–693. 368040 10.1128/jb.137.1.692-693.1979 PMC218506

[21] Osawa M, Anderson DE, Erickson HP (2008) Reconstitution of contractile FtsZ rings in liposomes. Science 320: 792–794. 18420899 10.1126/science.1154520 PMC2645864

[22] Hanahan D (1983) Studies on transformation of Escherichia coli with plasmids. J Mol Biol 166: 557–580. 6345791 10.1016/s0022-2836(83)80284-8

[23] Pla J, Sanchez M, Palacios P, Vicente M, Aldea M (1991) Preferential cytoplasmic location of FtsZ, a protein essential for *Escherichia coli* septation. Mol Microbiol 5: 1681–1686. 1943703 10.1111/j.1365-2958.1991.tb01915.x

[24] Casadaban MJ (1976) Transposition and fusion of the lac genes to selected promoters in Escherichia coli using bacteriophage lambda and Mu. J Mol Biol 104: 541–555. 781293 10.1016/0022-2836(76)90119-4

[25] Sambrook J, Russel D (2001) Molecular Cloning: A Laboratory Manual. Third Edition. Cold Spring Habour: Cold Spring Habour Laboratory Press.

[26] Pazos M, Natale P, Vicente M (2013) A specific role for the ZipA protein in cell division: stabilization of the FtsZ protein. J Biol Chem 288: 3219–3226. 23233671 10.1074/jbc.M112.434944 PMC3561543

[27] Raskin DM, de Boer PA (1997) The MinE ring: an FtsZ-independent cell structure required for selection of the correct division site in E. coli. Cell 91: 685–694. 9393861 10.1016/s0092-8674(00)80455-9

[28] Plá J, Palacios P, Sánchez M, Garrido T, Vicente M (1993) Stability of components of the *Escherichia coli* Septator. In: de Pedro MA, Höltje JV, Löffelhardt W, editors. Bacterial Growth and Lysis: Metabolism and Structure of the Bacterial Sacculus. New York: Plenum Press. pp. 363–368.

[29] Tsien RY (1998) The green fluorescent protein. Annu Rev Biochem 67: 509–544. 9759496 10.1146/annurev.biochem.67.1.509

[30] Vischer N Object J, Non-destructive marking and linked results in ImageJ, http://simon.bio.uva.nl/objectj/index.html (Accessed 2014 Feb 20). University of Amsterdam.

[31] Rasband WS (1997–2012) ImageJ-http://imagej.nih.gov/ij/ (Accessed 2014 Feb 20). U. S. National Institutes of Health, Bethesda, Maryland, USA.

[32] Addinall SG, Bi E, Lutkenhaus J (1996) FtsZ ring formation in *fts* mutants. J Bacteriol 178: 3877–3884. 8682793 10.1128/jb.178.13.3877-3884.1996 PMC232649

[33] Laemmli UK (1970) Cleavage of structural proteins during the assembly of the head of bacteriophage T4. Nature 227: 680–685. 5432063 10.1038/227680a0

[34] Towbin H, Staehelin T, Gordon J (1979) Electrophoretic transfer of proteins from polyacrylamide gels to nitrocellulose sheets: procedure and some applications. Proc Natl Acad Sci U S A 76: 4350–4354. 388439 10.1073/pnas.76.9.4350 PMC411572

[35] Lutkenhaus J (2007) Assembly dynamics of the bacterial MinCDE system and spatial regulation of the Z ring. Annu Rev Biochem 76: 539–562. 17328675 10.1146/annurev.biochem.75.103004.142652

[36] Ma L, King GF, Rothfield L (2004) Positioning of the MinE binding site on the MinD surface suggests a plausible mechanism for activation of the Escherichia coli MinD ATPase during division site selection. Mol Microbiol 54: 99–108. 15458408 10.1111/j.1365-2958.2004.04265.x

[37] Amann E, Brosius J, Ptashne M (1983) Vectors bearing a hybrid trp-lac promoter useful for regulated expression of cloned genes in Escherichia coli. Gene 25: 167. 6363212 10.1016/0378-1119(83)90222-6

[38] Weiss DS, Chen JC, Ghigo JM, Boyd D, Beckwith J (1999) Localization of FtsI (PBP3) to the septal ring requires its membrane anchor, the Z ring, FtsA, FtsQ, and FtsL. J Bacteriol 181: 508–520. 9882665 10.1128/jb.181.2.508-520.1999 PMC93405

[39] Ma X, Margolin W (1999) Genetic and functional analyses of the conserved C-terminal core domain of Escherichia coli FtsZ. JBacteriol 181: 7531–7544. 10601211 10.1128/jb.181.24.7531-7544.1999 PMC94211

[40] Osawa M, Erickson HP (2005) Probing the domain structure of FtsZ by random truncation and insertion of GFP. Microbiology 151: 4033–4043. 16339948 10.1099/mic.0.28219-0

[41] Pazos M, Natale P, Vicente M (2013) A Specific Role for the ZipA Protein in Cell Division: STABILIZATION OF THE FtsZ PROTEIN. J Biol Chem 288: 3219–3226. 23233671 10.1074/jbc.M112.434944 PMC3561543

[42] Mosyak L, Zhang Y, Glasfeld E, Haney S, Stahl M, et al (2000) The bacterial cell-division protein ZipA and its interaction with an FtsZ fragment revealed by X-ray crystallography. Embo J 19: 3179–3191. 10880432 10.1093/emboj/19.13.3179 PMC313961

[43] Szwedziak P, Wang Q, Freund SM, Lowe J (2012) FtsA forms actin-like protofilaments. EMBO J 31: 2249–2260. 22473211 10.1038/emboj.2012.76 PMC3364754

[44] Yu XC, Margolin W (1999) FtsZ ring clusters in min and partition mutants: role of both the Min system and the nucleoid in regulating FtsZ ring localization. Mol Microbiol 32: 315–326. 10231488 10.1046/j.1365-2958.1999.01351.x

[45] Mannik J, Wu F, Hol FJ, Bisicchia P, Sherratt DJ, et al (2012) Robustness and accuracy of cell division in Escherichia coli in diverse cell shapes. Proc Natl Acad Sci U S A 109: 6957–6962. 22509007 10.1073/pnas.1120854109 PMC3345019

[46] Cambridge J, Blinkova A, Magnan D, Bates D, Walker JR (2013) A replication-inhibited un-segregated nucleoid at mid-cell blocks Z-ring formation and cell division independently of SOS and the SlmA nucleoid occlusion protein in Escherichia coli. J Bacteriol. 10.1128/JB.01230-12 PMC3911132 24142249

[47] Potluri LP, de Pedro MA, Young KD (2012) Escherichia coli low-molecular-weight penicillin-binding proteins help orient septal FtsZ, and their absence leads to asymmetric cell division and branching. Mol Microbiol 84: 203–224. 22390731 10.1111/j.1365-2958.2012.08023.x PMC3323748

[48] Rodrigues CD, Harry EJ (2012) The Min system and nucleoid occlusion are not required for identifying the division site in Bacillus subtilis but ensure its efficient utilization. PLoS Genet 8: e1002561. 22457634 10.1371/journal.pgen.1002561 PMC3310732

[49] Si F, Busiek K, Margolin W, Sun SX (2013) Organization of FtsZ Filaments in the Bacterial Division Ring Measured from Polarized Fluorescence Microscopy. Biophys J 105: 1976–1986. 24209842 10.1016/j.bpj.2013.09.030 PMC3824704

[50] Sourjik V, Berg HC (2004) Functional interactions between receptors in bacterial chemotaxis. Nature 428: 437–441. 15042093 10.1038/nature02406

