# Correction: FtsZ Placement in Nucleoid-Free Bacteria

**DOI:** 10.1371/journal.pone.0101849

**Published:** 2014-06-26

**Authors:** The PLOS ONE Staff

There is an error in the citation only within the PDF of the published article. The last author in the citation should be Natale P. The correct citation is:

Pazos M, Casanova M, Palacios P, Margolin W, Natale P, et al. (2014) FtsZ Placement in Nucleoid-Free Bacteria. PLoS ONE 9(3): e91984. doi:10.1371/journal.pone.0091984
