# Supplementary figures and images for: FtsZ Placement in Nucleoid-Free Bacteria
Source: PLoS One. 2014 Mar 17;9(3):e91984. doi: 10.1371/journal.pone.0091984 (PMC3956765; doi:10.1371/journal.pone.0091984)

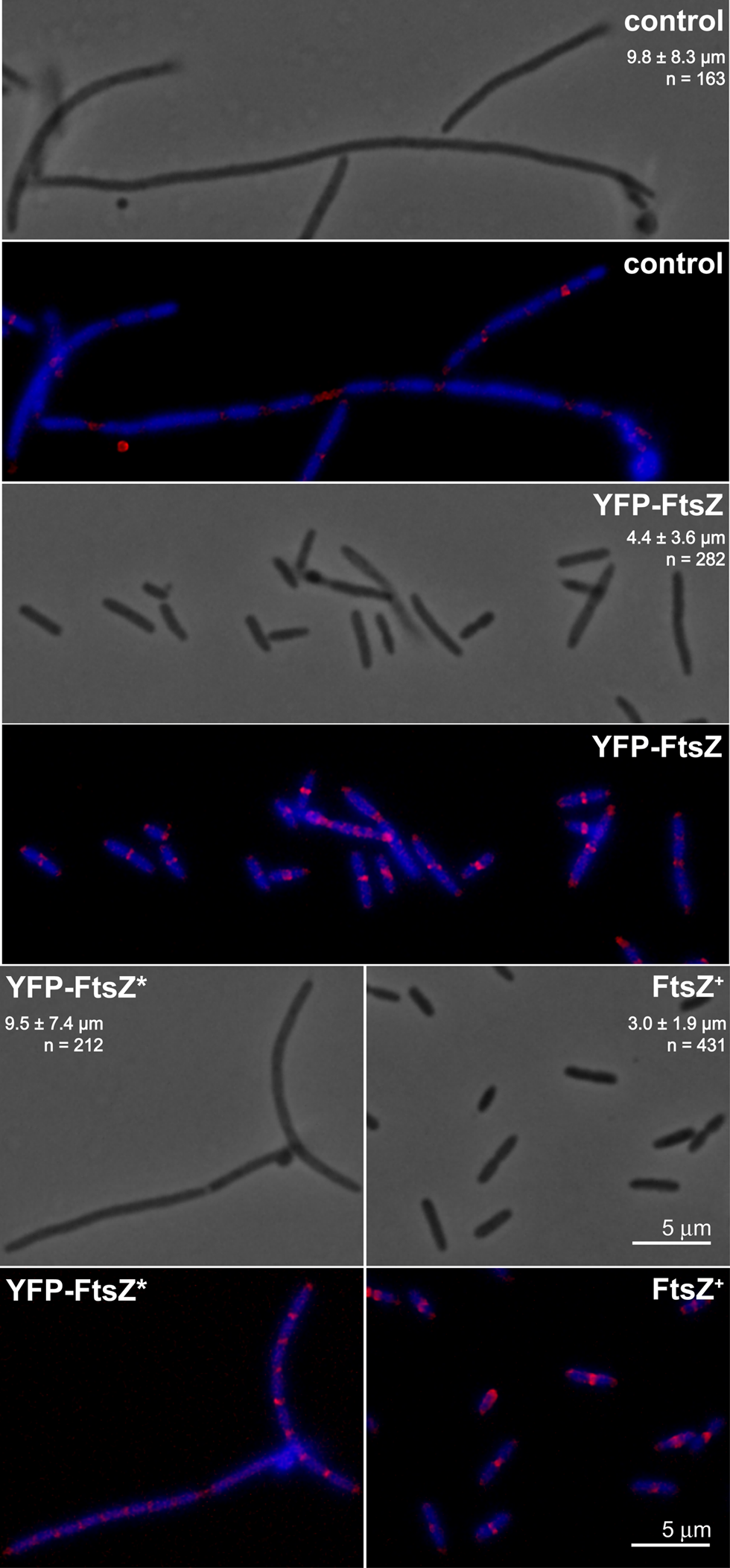

Supplement: Figure S1 — Immunolocalization of the plasmid-encoded FtsZ variants in FtsZ-depleted VIP2 cells. Cultures of VIP2 cells harbouring plasmids pPZV120 (control), pPZV110 (YFP-FtsZ), pPZV137 (YFP-FtsZ*) or pPZV138 (FtsZ+) growing exponentially at 30 °C were shifted to 42 °C and incubated for 180 min to deplete the amount of FtsZ+. Production of each plasmid-encoded FtsZ variant was then achieved by addition of IPTG (15 μM) during 150 min. Phase contrast and fluorescence merged micrographs of DAPI staining and FtsZ immunolocalization signal are shown for each strain. Mean cell length ± standart deviation (μm) and number of cells measured (n) are indicated in each case. (TIF) [file pone.0091984.s001.tif]

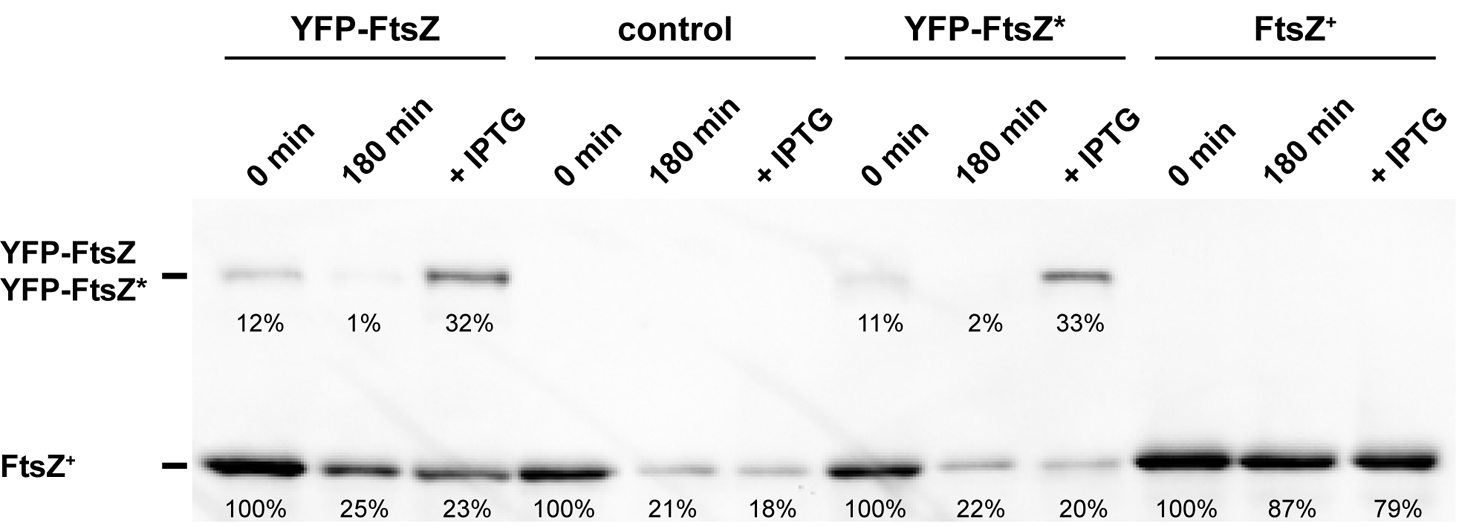

Supplement: Figure S2 — Production of FtsZ+, YFP-FtsZ and YFP-FtsZ* in FtsZ-depleted VIP2 cells. Immunoblot of extracts from VIP2 transformed with pPZV110 (YFP-FtsZ), pPZV120 (empty vector control), pPZV137 (YFP-FtsZ*) or pPZV138 (FtsZ+) revealed using MVC2 anti-FtsZ serum. The transformants were grown at 30 °C until they reached balanced exponential growth rate (lane: 0 min). At time 0 they were shifted to 42 °C and incubated for 180 min to deplete the amount of FtsZ+ (lane: 180 min). Production of each plasmid-encoded FtsZ variant was then achieved by addition of IPTG (15 μM) during 150 min (lane: +IPTG). The amount of FtsZ in each sample, relative to the amount of FtsZ present at 0 min (100%), is indicated below each lane. (TIF) [file pone.0091984.s002.tif]

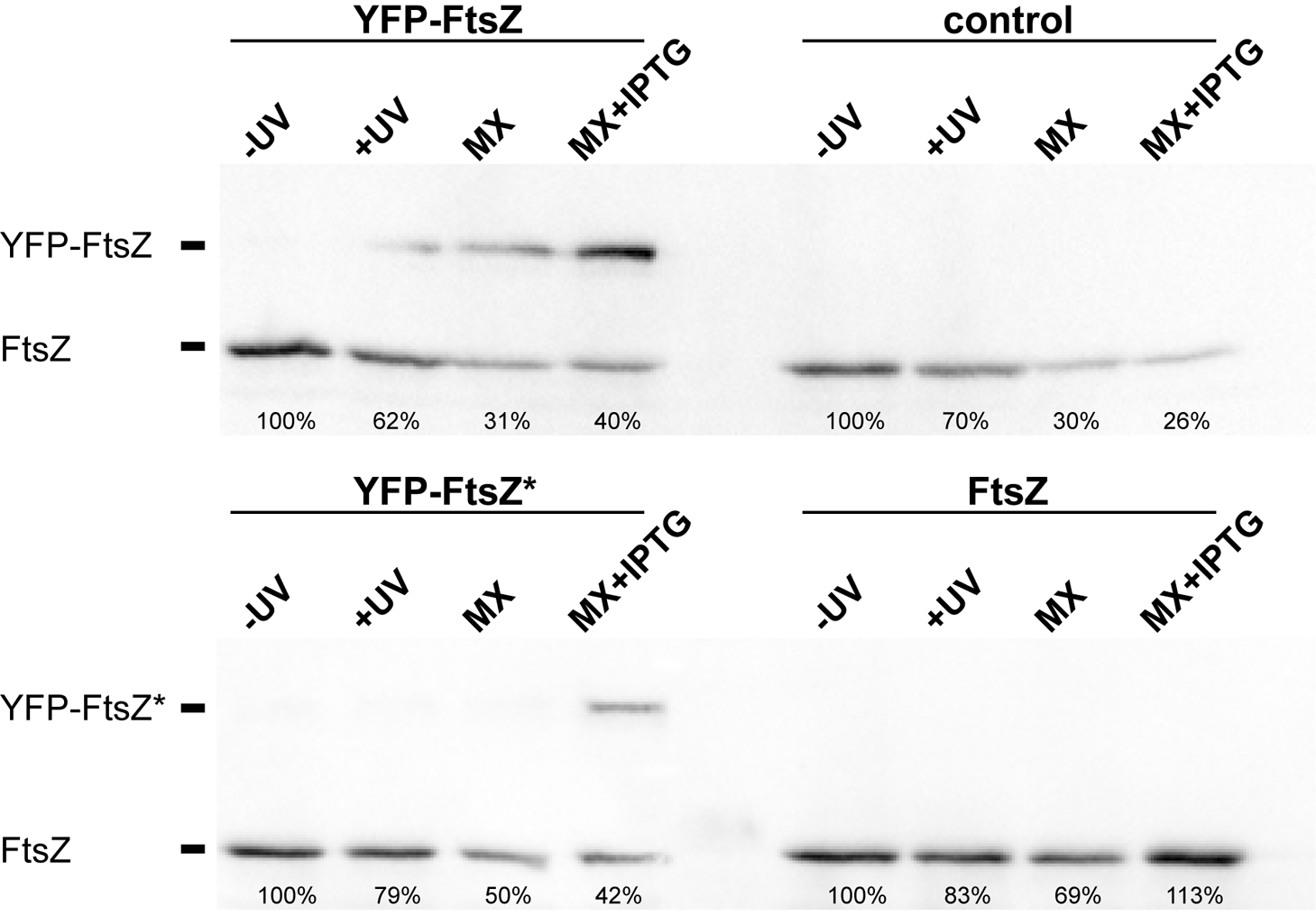

Supplement: Figure S3 — Production of FtsZ+, YFP-FtsZ and YFP-FtsZ* in maxicells. Western blot showing the protein level of the FtsZ variants during the maxicell procedure of E. coli CSR603 bearing pPZV110 (YFP-FtsZ), pDSW210 (empty vector control), pPZV137 (YFP-FtsZ*) or pPZV138 (FtsZ). (−UV) non-UV irradiated CSR603, (+UV) 3 hours after UV irradiation, (MX) 19 hours after irradiation and (MX+IPTG) 16 hours after addition of IPTG at time-point +UV. Relative amount of FtsZ is indicated below each sample. (TIF) [file pone.0091984.s003.tif]

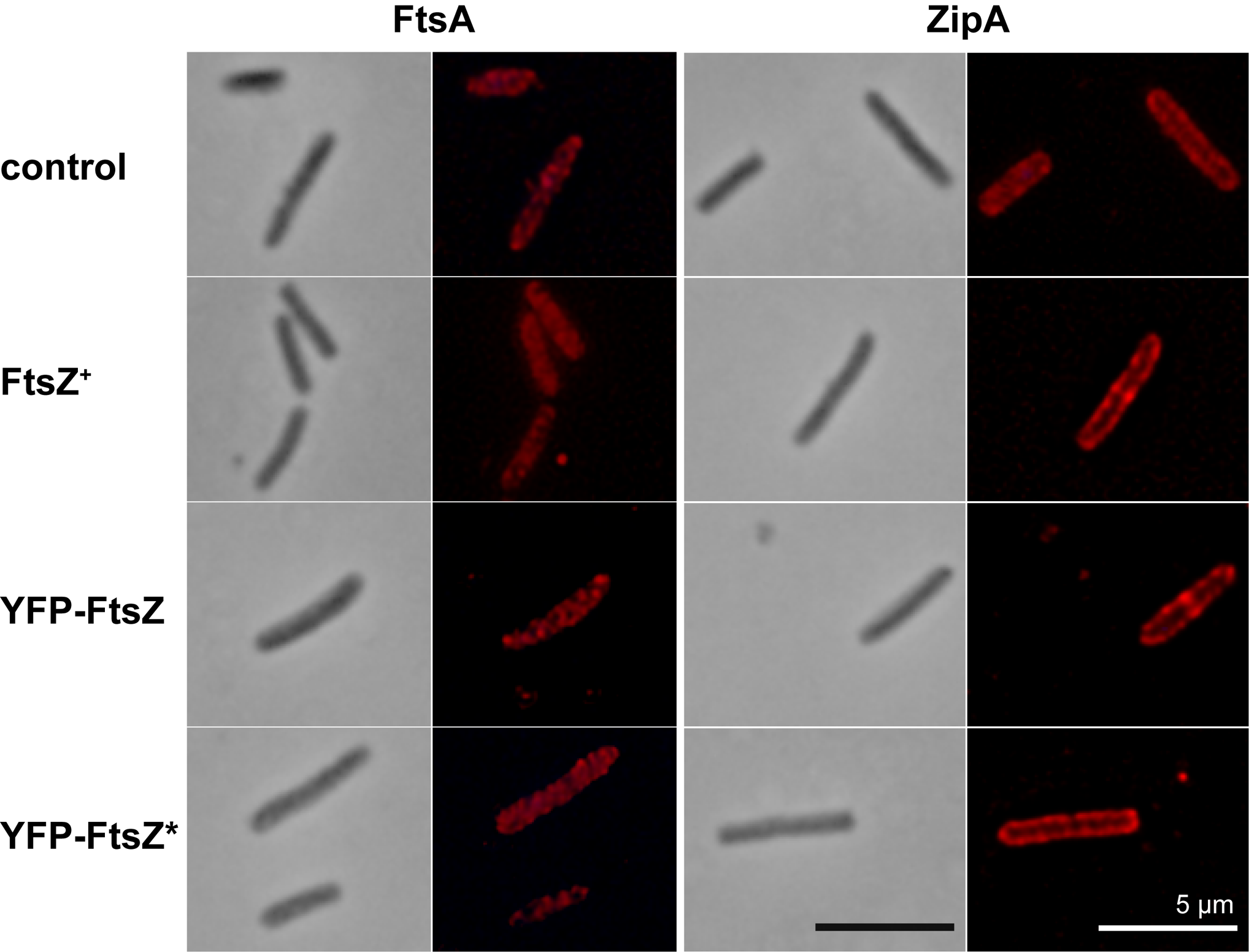

Supplement: Figure S4 — FtsA and ZipA localization in maxicells producing FtsZ variants. Phase contrast and fluorescence merged micrographs of DAPI staining and FtsA immunolocalization (left) or DAPI staining and ZipA immunolocalization (right). The images correspond to maxicells containing pPZV120 (control), pPZV138 (FtsZ+), pPZV110 (YFP-FtsZ) or pPZV137 (YFP-FtsZ*). These samples correspond to those analyzed by Western blot in lane MX+IPTG in Figure S3 (see Figure S3 legend). (TIF) [file pone.0091984.s004.tif]

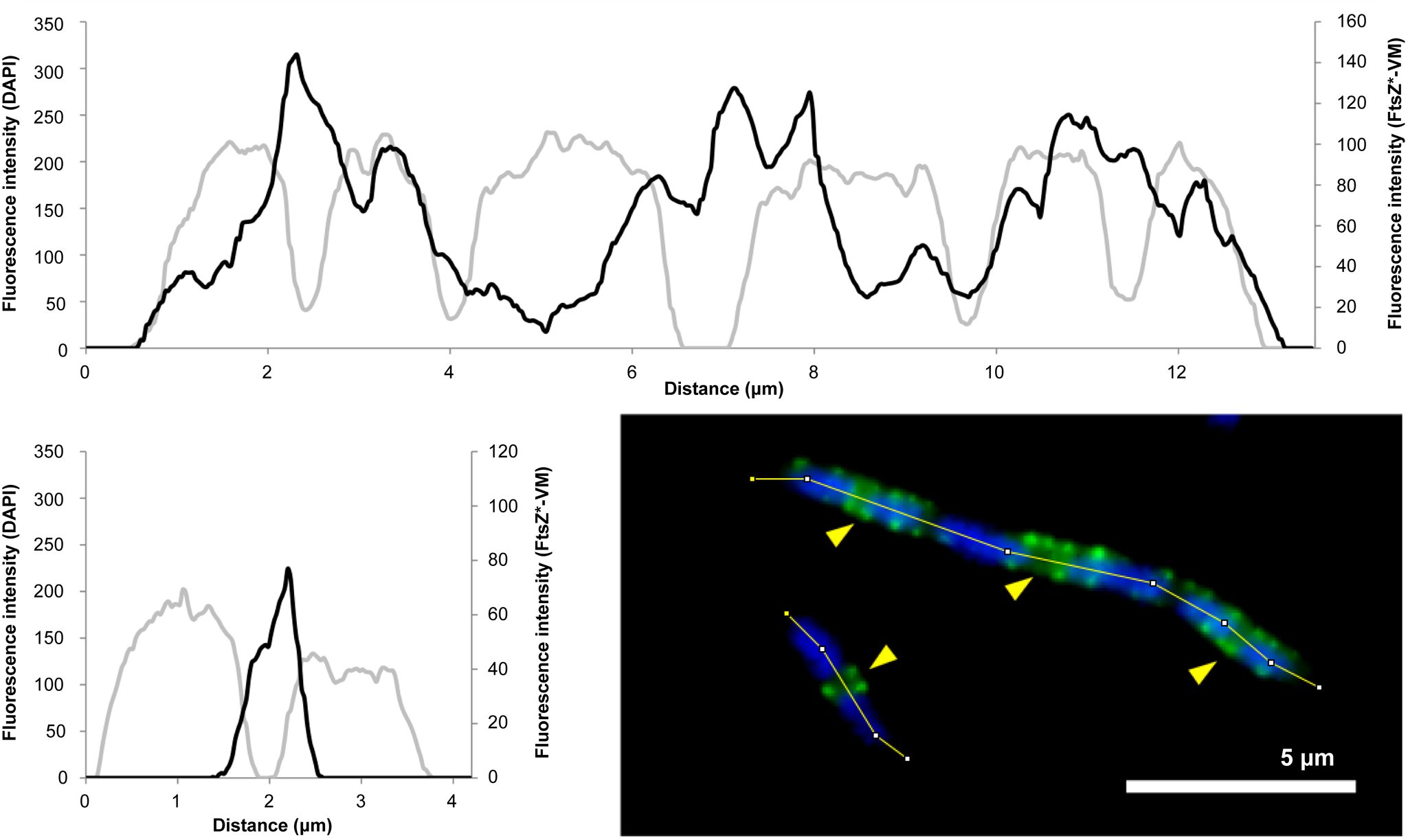

Supplement: Figure S5 — Localization of FtsZ*-VM in FtsZ-depleted VIP2 cells. Line profiles of fluorescent signals emanating from the cells shown in Figure 5C. Arbitrary fluorescent units are plotted on the y axis and cell length (in μm) is plotted on the x axis. Grey lines correspond to DAPI staining (nucleoids position) and black lines correspond to FtsZ*-VM fluorescence. (TIF) [file pone.0091984.s005.tif]
