# Supplementary material for: FtsZ Placement in Nucleoid-Free Bacteria
Source: PLoS One. 2014 Mar 17;9(3):e91984. doi: 10.1371/journal.pone.0091984 (PMC3956765; doi:10.1371/journal.pone.0091984)
Supplement: Table S1 — Oligonucleotide primers used in this study. (DOC) [file pone.0091984.s006.doc]

**Table S1. Oligonucleotide primers used in this study**

| **Primers** | **Orientation^a^** | **5′→3′ Sequence** | **Target** |
| --- | --- | --- | --- |
| 1455 | Fw | AAAAAACTAGTATGTTTGAACCAATGGAACTTACC | *ftsZ* |
| 1469 | Rv | ATATAGAGCTCAAGCTTTTAATCAGCTTGCTTACGCAGGAA | *ftsZ* |
| 1478 | Fw | ACAATGCATGGATCCatgagtaaaggagaagaac | *yfp* |
| 1491 | Rv | CACAACTAGTTTTGTATAGTTCATCCATGCC | *yfp* |
| 1503 | Fw | CACAGCGGCCGCTCTAGAATGAGTAAAGGAGAAGAACTTTTC | *yfp* |
| MP17 | Rv | CCCAAGCTTGTTATCCTCCGAACAAGCGTTTGAGGAAGC | *minD* |
| MP18 | Fw | CCCAAGCTTGGATCCATGTTTGAACCAATGGAACTTACCAATGAC | *ftsZ* |
| MP34 | Fw | AACATATGGCATTACTCGATTTCTTTCTC | *minE* |
| MP35 | Rv | TTTGAATTCCTTATTTCAGCTCTTCTGC | *minE* |
| MP42 | Rv | ATATAGAGCTCAAGCTTTTACGCAGTTTGCGGCGCATTGTCATTC | *ftsZ* |
| MP43 | Fw | AAAAATCTAGAATGAGTAAAGGAGAAGAACTTTTC | *yfp* |
| MP44 | Fw | AAAAATCTAGAATGTTTGAACCAATGGAACTTACC | *ftsZ* |
| MP45 | Rv | ATATAGAGCTCAAGCTTTTAATCAGCTTGCTTACGCAGG | *ftsZ* |

^a^ Fw: forward, Rv: reverse
