# Supplementary material for: FtsZ Placement in Nucleoid-Free Bacteria
Source: PLoS One. 2014 Mar 17;9(3):e91984. doi: 10.1371/journal.pone.0091984 (PMC3956765; doi:10.1371/journal.pone.0091984)
Supplement: Text S1 — Supporting Material and References. (DOC) [file pone.0091984.s007.doc]

**Supporting Material For:**

**Selection of FtsZ placement in nucleoid-free bacterial**

**Manuel Pazos^1^, Mercedes Casanova^1^, Pilar Palacios^1^, William Margolin^2^, Paolo Natale^1^ and Miguel Vicente^1^***

**^1^** Centro Nacional de Biotecnología - Consejo Superior de Investigaciones Científicas (CNB-CSIC), 28049 Madrid, Spain

**^2^** Department of Microbiology and Molecular Genetics, University of Texas Medical School at Houston, Houston, Texas, USA

* To whom correspondence should be addressed: Miguel Vicente, Centro Nacional de Biotecnología (CNB-CSIC), C/ Darwin 3, 28049 Madrid, Spain, Tel. +3491 585 46 99, Fax. +3491 585 45 06; E-mail: mvicente@cnb.csic.es

**MATERIAL AND METHODS**

**Production of anti His-MinE antibody.** Plasmid pPZV46 was constructed, where a *minE* PCR product was cloned into the pET28a expression vector (Novagen) and in this way fused to an amino-terminal hexa-histidine tag. The *minE* PCR fragment was obtained by standard PCR using oligonucleotide primers MP34 and MP35, *Taq* polymerase (Roche) and chromosomal DNA as template with an annealing temperature of 55ºC. The chromosomal DNA of *E. coli* MC4100 [1] was isolated according to Baele *et al.* [2]. His-MinE was overproduced and purified according to the pET-vector expression systems in combination with nickel-affinity chromatography (His-Bind Resin) according to manufacturers manual (Novagen Biochemicals). Purified His-MinE was electroblotted on a nylon blotting membrane (Roche) and send for the production of polyclonal anti-His-MinE containing rabbit blood serum (MVZ1) (PickCell Laboratories BV, Lelystad, The Netherlands).

**REFERENCES**

1. Casadaban MJ (1976) Regulation of the regulatory gene for the arabinose pathway, araC. J Mol Biol 104: 557-566.

2. Baele M, Devriese LA, Haesebrouck F (2001) Lactobacillus agilis is an important component of the pigeon crop flora. J Appl Microbiol 91: 488-491.
